# Comparative Associations of Street Network Design, Streetscape Attributes and Land-Use Characteristics on Pedestrian Flows in Peripheral Neighbourhoods

**DOI:** 10.3390/ijerph16101846

**Published:** 2019-05-24

**Authors:** Ayse Ozbil, Tugce Gurleyen, Demet Yesiltepe, Ezgi Zunbuloglu

**Affiliations:** 1 Department of Architecture and Built Environment, Northumbria University, Newcastle NE1 8ST, UK; demet.yesiltepe@northumbria.ac.uk; 2 Department of City and Regional Planning, Istanbul Technical University, Istanbul 34367, Turkey; tugcegurleyen@gmail.com; 3 Department of Urban Design, Istanbul Technical University, Istanbul 34367, Turkey; ezgi.zunbuloglu@gmail.com

**Keywords:** street network configuration, peripheral neighbourhoods, pedestrian flow, streetscape features, Istanbul

## Abstract

Research has sufficiently documented the built environment correlates of walking. However, evidence is limited in investigating the comparative associations of micro- (streetscape features) and macro-level (street network design and land-use) environmental measures with pedestrian movement. This study explores the relative association of street-level design-local qualities of street environment-, street network configuration –spatial structure of the urban grid- and land-use patterns with the distribution of pedestrian flows in peripheral neighbourhoods. Street design attributes and ground-floor land-uses are obtained through field surveys while street network configuration is evaluated through space syntax measures. The statistical models indicate that the overall spatial configuration of street network proves to be a stronger correlate of walking than local street-level attributes while only average sidewalk width appears to be a significant correlate of walking among the streetscape measures. However, the most significant and consistent correlate of the distribution of flows is the number of recreational uses at the segment-level. This study contributes to the literature by offering insights into the comparative roles of urban design qualities of the street environment and street network layout on pedestrian movement. The findings also offer evidence-based strategies to inform specific urban design and urban master planning decisions (i.e., the provision of more generous sidewalks on streets with relatively higher directional accessibility) in creating lively, walkable environments.

## 1. Introduction

Physical activity is an important lifestyle component of improving long-term health [1]. Walking is the most common form of adult physical activity [1,2]. Earlier studies point to the positive effects of walking on various government priorities including but not limited to, air quality and pollution [3], physical activity, obesity [4], mental health [5] and congestion [6]. Research indicates that walking reduces anxiety, depression, anger and time pressure [7] tension and confusion [8] and increases creativity [9]. Moreover, studies also argue that walking and cycling reduces health and parking costs [10]. Hence, developing walkable environments is key in promoting sustainable urban neighbourhoods [11,12]. 

Researchers and practitioners alike agree on the importance of the built environment in facilitating or restraining walking. Hence, it is important to understand the built environment correlates of walking to provide an empirical basis for planning and urban design actions aimed at creating walkable environments. A growing body of research relates pedestrian-friendly neighbourhood design to measured walking behaviour [13,14]. The underlying idea is that environmental supports for physical activity will enable people to walk more and thereby be more active. Yet few neighbourhood studies actually include the micro-scale (i.e., the presence and continuity of sidewalks) and the macro-scale (i.e., spatial structure of urban networks) environmental features in the same model. Hence, the present research is designed to (a) identify the extent to which micro-scale (street-level urban design qualities) and macro-scale (street network configuration and land-use) environmental correlates are associated with pedestrian movement and (b) consider the implications of the findings for urban design and planning-policies aimed to design active built environments.

### 1.1. Built Environment Correlates of Walking

The past decade has witnessed a growing attention to the physical environmental correlates [15,16,17,18] of walking behaviour. Previous reviews on children, adolescents and adults have reported consistent relationships between physical environmental characteristics and physical activity, in particular walking [19,20,21]. Studies that examined associations between attributes of the built environment and walking have focused on the macro-scale (land-use patterns and street network design) and micro-scale (street design) environmental characteristics.

### 1.2. Macro-Scale Environmental Correlates

#### 1.2.1. Land-Use Characteristics 

The systematic reviews on studies investigating the empirical analysis of macro-scale environmental correlates of walking have demonstrated consistent positive relations between walking and land-use mix (proximity of homes and destinations such as shops) [22,23,24]. These reviews and more concluded that mixed land-use, which is due to the decreased distance between or intermingling among different types of land uses, such as residential and commercial uses, is associated with more walking. It is argued that mixing offices, shops, restaurants, residences and other activities influences the decision to walk by making it more convenient to walk to various destinations [25,26] while having destinations within walking distance from origins (homes, stations, schools, etc.) increases the odds of walking [27,28]. This finding is also related to compactness (or density) of land-uses. In areas with higher density of land-uses, destinations can be closer together, which is thought to shape pedestrian activity by bringing numerous activities closer together, thus increasing their accessibility from trip origins [13,29]. It is suggested that people are willing to use slower modes of travel, such as walking, for shorter distances, especially if many trips can be chained [30,31]. Although the literature has demonstrated a strong positive relationship between non-residential uses and walking in general, the conclusions regarding the impacts of recreational uses on pedestrian movement is ambiguous. While some studies could not identify public open space (e.g., parks) as a significant correlate of walking for leisure or transport [32,33], others demonstrated that open space was positively related to walking for transport but not walking for recreation [34]. 

#### 1.2.2. Street Network Design

Researchers in transportation and planning have also reported consistent relationships between street network design and walking behaviour [22,35]. The extent to which different parts of a neighbourhood are linked to one another determines the level of street connectivity. Here street connectivity refers to how connectivity is measured in general, not limiting to how it is correlated with pedestrian movement. Street connectivity refers to the degree to which pedestrian movement can flow with ease and it provides multiple choices between any two locations [36]. It can be measured with percent of gridded streets in a buffer of a person’s home [37,38], distance between intersections [39,40], directness of routes [41,42], the area-weighted average perimeter [43] or “network density” [44]. Some researchers have used walkable catchments or “pedsheds” to measure the accessible streets along the network [45,46,47]. After discussing the shortcomings of existing measures of permeability, which relate to the capacity to move and the potential to interact in an urban environment, Pafka and Dovey [48] introduced “area-weighted average perimeter” and “interface catchments” as more effective measures of permeability that can measure both walkable access and what one gets access to. The commonly used block length measure denotes the average street segment length within an area and that several variants of block size have been used including block perimeter, block area and block density [49,50,51]. Criticism of several popular connectivity measures, such as intersection density (number of intersections per given area) and block length (street segment length), have revealed significant flaws in these measures [43,52,53]. Stangl and Guinn [54] argue that intersection density measures fail to account for street pattern and its actual permeability and that movement may be completely obstructed in areas with good intersection density scores. Route directness, which is the ratio of the shortest distance between two points on a network to the straight-line distance between these points, has also been applied, though less frequently, to measure the ease of movement to a destination (i.e., school) [41,47,55]. To make this measure more applicable to area-wide connectivity assessment, Stangl and Guinn [54] and Stangl [56] adapted a modified route directness measure, which can directly assess permeability.

In order to encourage non-motorized travel, continuous non-motorized rights of way must be provided that allow pedestrians to reach various destinations within a city [57]. Continuous street pattern not only reduces trip length, but it also offers greater choice of travel routes and modes. Higher street connectivity related to increased walking is defined as increased number of intersections with fewer dead-end streets [58], more streets [59], high node-link ratio [60] and smaller block length [61]. Street patterns with gridded street networks, which tend to have relatively higher street connectivity and street network density, are associated with increased walking and biking [62]. The 1 km Euclidean buffer was determined as the easy-walking distance [63] and used by studies in order to capture pedestrian activity in neighbourhoods [64,65]. Higher intersection density and link-node ratio within 1 km Euclidean buffers of homes were found to be [66] related to increased frequency of walking. Similarly, studies measuring connectivity at the neighbourhood level, found a positive relationship with total walking [67,68].

Even though research investigating the influences of land-use and street connectivity on walking has proliferated in recent years, no conclusions emerge on the relationships between street network design and travel. Part of the reason is due to collinearity between land-use mix and street network design. Fairly compact neighbourhoods, particularly in US and Australian cities, generally have more varied land-uses, on average shorter block lengths with more grid-like street patterns. Thus, the effect of street network design on overall travel remains unclear. Another reason is that the above-described measures describe the average connectivity properties of street networks. However, they fall short in describing the spatial and structural pattern of street networks that define urban areas. 

The significance of the spatial structure of street networks in explaining walking behaviour has been apparent in recent studies [69,70,71]. Spatial structure may be defined as the collection of streets and street segments through certain alignments and hierarchies. The significance of spatial structure as a crucial correlate of walking has been highlighted within space syntax theory. Space syntax is a set of techniques that is used to better understand the interaction between societies and the spaces [72]. The main idea in space syntax is that not the buildings but the spaces between buildings –not solids but voids– are important as these are the spaces where people interact [73]. At the urban scale, it is a tool to describe and quantitatively measure the spatial configuration of public spaces, that is, street systems. Evidence from studies applying space syntax methodology suggests that streets that are accessible from their surroundings with fewer direction changes (evaluated through the connectivity measure of Integration) tend to attract higher densities of pedestrian flows [74,75]. Drawing on the work of space syntax, some researchers have applied metric reach, which measures the amount of street length accessible within a specific walking distance from the centre of each street segment in an urban network [76], to show that the configuration of individual street elements within an area is significantly associated with walking [47,77]. Moreover, recent studies have also shown that the structure of an urban street network, as defined by the connectivity hierarchy measured by direction changes (through a recent connectivity measure of directional reach), has an important impact on pedestrian travel [77,78].

### 1.3. Micro-Scale Environmental Correlates

Studies investigating the environmental correlates of walking have sufficiently documented associations between micro-scale environmental attributes (pedestrian-friendly street design) and pedestrian activity. In fact, some researchers argue that in spite of the plethora of studies on macro-level urban form characteristics, studies focusing on micro-level attributes are limited [79,80]. In related literature, street-level walkability indicators that affect pedestrian experience include pedestrian-oriented design features, such as pedestrian crossings (e.g., pedestrian crossing coverage rate, signal coverage rate and crossing facility design index), sidewalks (e.g., sidewalk coverage rate, sidewalk width, length) as well as curb to curb roadways (e.g., number of traffic lanes). Indeed, surveys of the literature found sufficient evidence to conclude that the continuity and width of sidewalks [21,81,82], the presence and ease of street crossings [83], aesthetic qualities (the attractiveness of the environment, presence of tree-lined streets) [84] and signalization [39,85,86,87,88], as well as the presence of aesthetic or safety features, such as cleanliness, interesting sights and architecture [34,89,90], encourage walking among adults and children. For example, by conducting a Delphi study with experts, Pikora et al. [39] identified five factors among a list of potentially important environmental factors: safety, aesthetics, destination, functionality and subjective assessments. This study underlined the impact of micro-scale criteria, such as crossings, path continuity (for safety), presence of trees, parks, maintenance and cleanliness (for aesthetics) and attractiveness and difficulty of the environment (for subjective assessments), on walking. Wilcox and others [91] defined nine environmental factors in their study related to physical activity in the US. They mentioned the importance of the presence of sidewalks, effect of heavy traffic, hills, presence of streetlights, having an enjoyable scenery, crime rates, observing others exercising and accessibility of walking trails to analyse environmental characteristics. In their audit comparison chart, Lee and Talen [92] stated four key factors: land-uses, sidewalks, vehicle-pedestrian interactions and safety and appeal. In this study, researchers listed the quality of sidewalks (aesthetics), natural barriers (ditch or creeks), unique markers, enclosure as criteria, different from other research. Bentley et al. [93] showed that increasing proportion of segments with a walking/cycling path-design and proportion of streets with one or more crossings were associated with more time spent on walking. However, in their study, which included crossing aids and trees as part of the street-level attributes to calculate an Environmental Factor Score, Pikora et al. [94] did not find any significant relationship between these factors and walking behaviour. In some studies using mixed-methods, participants reported access to sidewalks as key characteristics that support their walking [95,96], while lack of pedestrian crossings were reported to be a barrier [97]. Effect of sidewalks, safety, lighting, recreational facilities was discussed by others researchers as well [98,99]. In accordance with the results demonstrated in literature, urban planning and transport policies employ several strategies, such as providing pedestrian crossing devices, to improve the safety of pedestrians and thus, to encourage walking [100,101,102]. 

Although there is much evidence documenting the relationship between the built environment and walking, their usefulness is limited for urban designers and planners working at the local, neighbourhood level. One of the underlying reasons is that most of the indicators applied in the literature account for relatively coarse-resolution data, such as census tract levels, which do not lend to any useful findings for local comparative analysis. Second, to help policy makers and planners/designers in the assessment of urban areas, these analyses need to be localised but related studies generally focus on a comparison between cities rather than within cities. Third, the indicators used in such studies fail to evaluate spatial structure of urban form. For example, although most studies aim to evaluate the built environment on the basis of land-use patterns and street-level attributes, they tend to omit qualities regarding the spatial configuration of street networks. Lastly, while there is substantial work on the relationship between urban form (land-use and street design) and walking, both the transportation and physical activity literatures largely ignore the micro-scale environmental correlates deemed so important by urban designers. More importantly, only few studies contain objective measures of streetscape design quality [103,104]. 

To address the above-mentioned limitations of current studies, we examined the relative associations of macro-scale (land-use patterns and street network configuration) and micro-scale (local qualities of street environment) built environment correlates of walking with the observed distribution of pedestrian flows in four peripheral neighbourhoods in İstanbul. In doing so, we were able to develop well-specified statistical models that allow researchers to accurately evaluate the individual effects of each variable. Additionally, the quantitative data applied in this study is based on a smaller unit of assessment (street segment). Hence, this study gauges the significance of fine-grained design features that are fundamental for urban designers. Finally, in order to localise the findings in a meaningful way, this study focuses on an intra-urban (within city) comparison. Thus, the present research relates one walking-friendly neighbourhood environmental indicator –quantitative evidence of pedestrian movement– to objective macro- and micro-scale measures of the built environment. Based on this quantitative comparison some practical design guidelines are suggested towards more pedestrian-friendly peripheral districts.

## 2. Design of the Study/Method

### 2.1. Case Context

**Figure 1:**
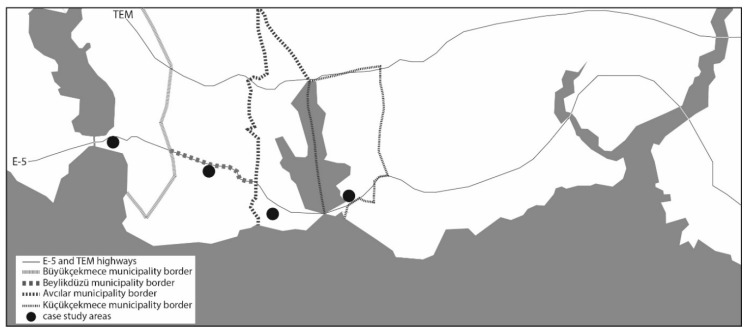
The location of case study areas within the city map.

Case studies of this paper are chosen from İstanbul’s peripheral areas, which function as sub-centres for their surroundings. These neighbourhoods—Küçükçekmece, Avcılar, Büyükçekmece and Beylikdüzü—are located in districts which have grown towards the periphery after the 1980s, parallel to E-5 highway, and have dominated the macro-form of the city (Figure 1). The selected areas are directly related to E-5 highway, which served as pedestrian access for the integration of the increased populations agglomerated within peripheral neighbourhoods with the sub-centres. These neighbourhoods are also directly related to the existing Bus Rapid Transit (BRT) line and its stations along the E-5 highway. This transit network, which was integrated into the mass rapid transit system of the city in 2007, has generated significant transit links that help integrate the peripheral populations with the study areas. Each area varies in the spatial configuration of its surrounding urban fabric, differing in the layout of street networks, morphological characteristics and land-use compositions, while all of them have similar characteristics (socio-economic and demographic structure). 

Avcılar consists of an urban fabric that is made up of low-rise (3-4 storeys) buildings built on the perimeter of the block. The buildings are vertically mixed-use (non-residential uses located on the ground floor, residences on the upper floors). The uniform urban grid is clear with regular urban blocks, with average size of 90 by 100 m. It includes a fine-grained land-use pattern, with small shops, cafes and so forth, spread evenly within the neighbourhood. 

While Beylikdüzü has similar average number of retail activities on the ground-floor as Avcılar, the first encompasses coarse-grained active ground floor uses (i.e. large shopping malls). The urban fabric in Beylikdüzü comprises a mixed street layout pattern: partial grid-iron layout with relatively larger blocks (150 by 200 m) as well as a partial curvilinear pattern. Individual high-rise blocks are located sparsely within the urban blocks. 

Küçükçekmece neighbourhood is characterized by a dominating curvilinear street network pattern, which partially turns into cul-de-sacs, with varied block sizes and lot patterns. The average block size is 100 by 200 m. As opposed to Beylikdüzü, the blocks in Küçükçekmece are relatively densified with buildings of relatively smaller plots (30 × 50 m). The urban fabric has relatively higher commercial street fronts.

Büyükçekmece neighbourhood is a predominantly residential neighbourhood at the ground-floor level. The urban fabric is characterized by partially a deformed urban grid-iron and partially a curvilinear street network pattern with varied block sizes and lot patterns. The average block size within the grid-iron part is 150 × 200 m whereas it is 100 × 100 m within the curvilinear system.

### 2.2. Methodology

#### 2.2.1. Pedestrian Observations

**Figure 2:**
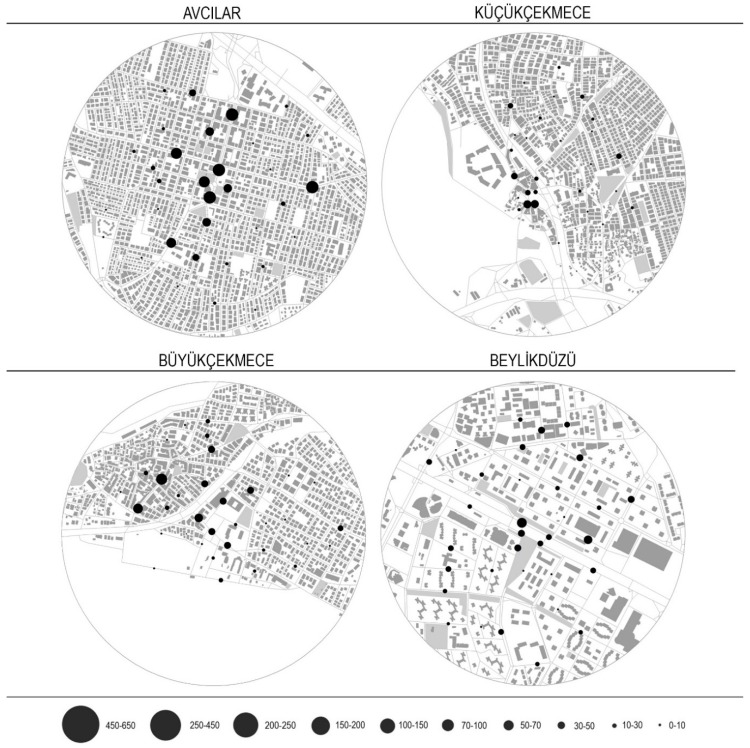
Graphic representation of observed pedestrian densities in four areas.

**Figure 3:**
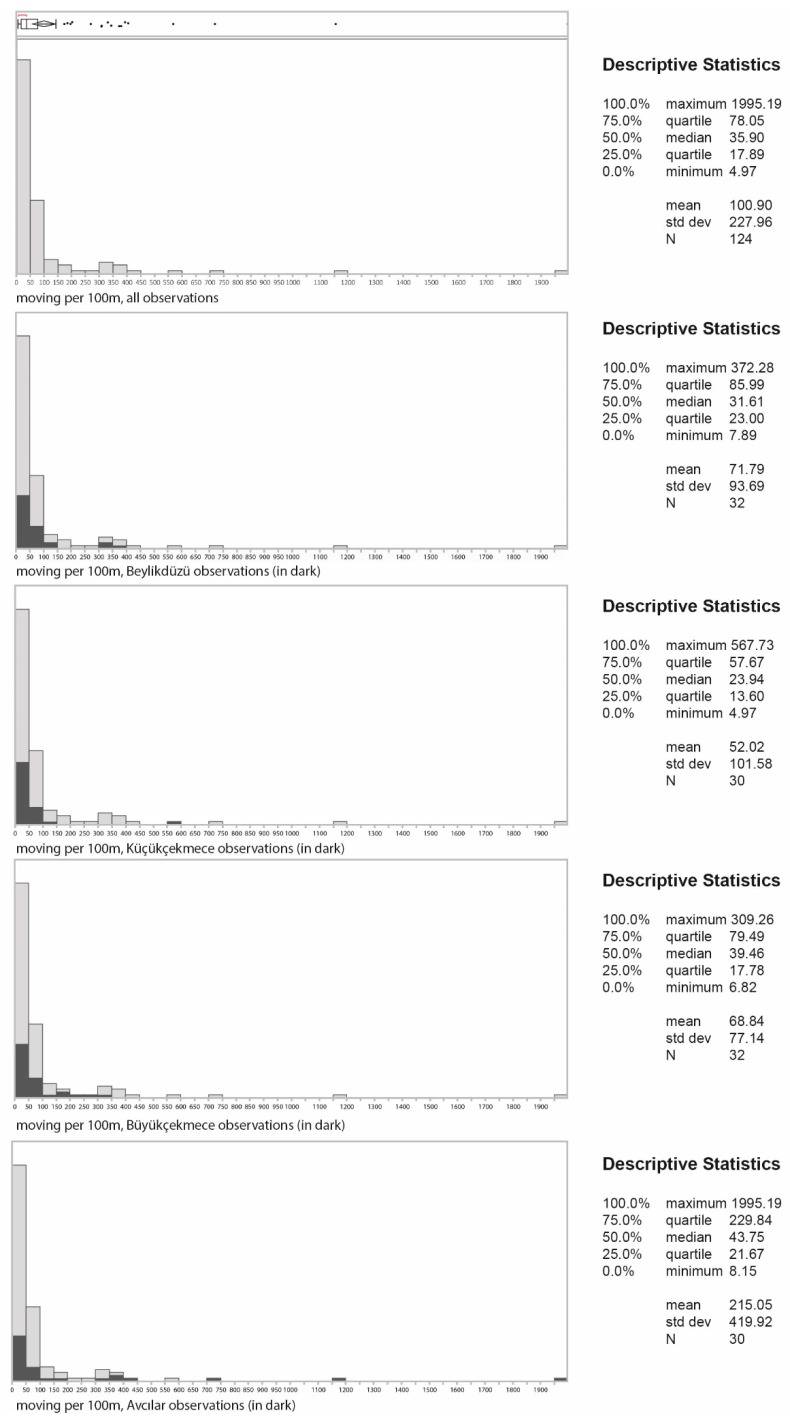
Statistical profile of observed pedestrian densities.

Pedestrian counts were collected on street segments within four neighbourhoods in İstanbul. The case context borders were designated as 800 m buffers surrounding the public square within each neighbourhood. 800 m distance was selected as the threshold since guidelines often use one-half mile (800 m) as a key distance in network planning [105,106]. Due to resource limitations, approximately 30 street segments were observed per area. These segments were selected to include a variety of connectivity levels, measured through Integration. Integration is a structural connectivity measure which calculates how close each segment is to all the others within a radius. Proportionate stratified random sampling was applied to select the segments. Street segments located within the study areas were grouped as low (bottom tercile), medium (middle tercile) and high (top tercile). Terciles are identified based on the Integration values for each street segment. Similar numbers of segments (~30) from each category was randomly selected to measure pedestrian flows. Pedestrian observations were conducted for 10-min intervals on two different weekdays distributed over two different time periods (morning and afternoon) per day. 10-min interval is used since this duration appears to be the length of manual count which is most commonly applied in literature [107,108,109]. Pedestrian counts were observed on weekdays only, since preliminary observations indicated no significant differences between weekday and weekend pedestrian activity rates within the areas. Figure 2 illustrates graphically the distribution of movement densities using circles of different diameters for the selected areas. Figure 3, which provides statistical information on pedestrian densities, shows how strongly the four areas differ. The median density of moving pedestrians per 100 m is 31.6, 23.9, 39.5 and 43.8 for Beylikdüzü, Küçükçekmece, Büyükçekmece and Avcılar respectively, while the corresponding means are 71.8, 52.0, 68.8 and 215.1. In total 124 street segments were observed. 

Ethics approval was granted by Human Ethics Commission, Özyeğin University (Ethics ID 2015/01) and relevant permissions were granted by the İstanbul Metropolitan Municipality (ID 30872936-02-622.1-1768-42338).

#### 2.2.2. Street Design and Land-Use

The same street segments selected for pedestrian observations were characterised through detailed field surveys to document the street-level pedestrian environment. The pedestrian quality attributes to document were selected from local qualities of street environment that are shown to affect pedestrian movement behaviour via their impacts on people’s perception on safety and aesthetics [39,110,111,112,113,114]. These include average sidewalk width as well as the presence of pedestrian crossings, traffic lights and trees. Where available, sidewalk width on both sides of the segment was measured and the average width is included in the analysis. Similarly, the presence of trees for both sidewalks is considered (i.e., coded “yes” if there were trees on either side of the audited segment). The selected segments were also surveyed in terms of the number of ground-floor frontages opening directly onto the street, relativized by street length. Land-use was categorised into residential, retail (including commercial and offices) and recreational (i.e., public parks and open areas for recreation such as playgrounds) to distinguish between the effects of each on the distribution of movement.

#### 2.2.3. Street Network Configuration

**Figure 4:**
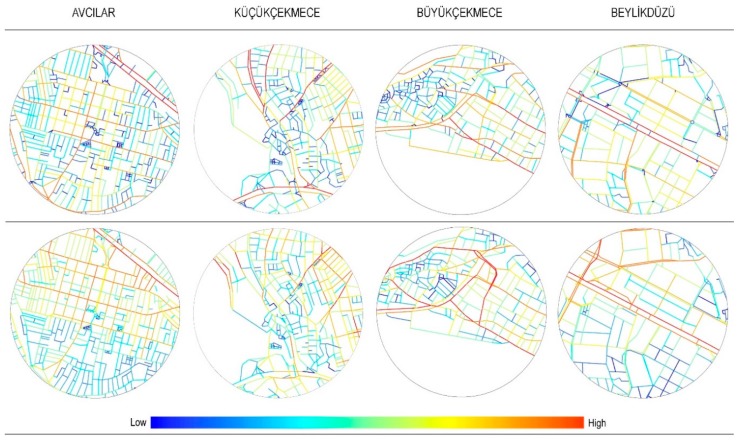
Street network configuration within study sites, coded according to: top) Directional reach (2-direction changes, 20°) and bottom) Integration (r:n).

To assess the street network configuration within the study areas, the entire street network of the European part of İstanbul Metropolitan area was evaluated using a standard space syntax measure, angular segment Integration and a more recent segment-based syntactic measure, Directional Reach. Integration measures how accessible each space is from all the others within the radius using the least angle measure of distance. Directional reach measures the total street length accessible within a specific number of direction changes from the centre of each street segment in an urban network. Directional reach was computed for 2 direction changes subject to a 20° angle threshold. Computing directional reach for two direction changes provides an estimate of how well a street segment is embedded in its surroundings from the point of view of directional distance. 20° was selected as the threshold since it reveals continuities that correspond to named streets and also in the sense that it helps identify stronger associations between street connectivity and non-residential land-uses, as well as stronger associations between street connectivity and vehicular traffic [115]. Figure 4 illustrates the street network configuration of each study area embedded within the surrounding 800 m radius buffer, coded according to Directional reach (2-direction changes, 20^o^) and Integration (n). Integration radius n, (n), is a global measure, which calculates the distance from each segment to all the others within the system. Hence, it represents the integration pattern of a system at the largest scale. 

Integration was calculated using Depthmap software [116,117], while Directional Reach was calculated in Java. Figure 4 illustrates study area street networks using Integration and Directional Reach. Streetmap 2014 obtained from the İstanbul Metropolitan Municipality was used to calculate these different street connectivity measures. ArcGIS 10.2.2 (Geographic Information Systems) (ESRI, 2014, Redlands, CA, USA) was used to merge all these different data sets. Linear models were developed in JMP (JMP®, Version 13. SAS Institute Inc., Cary, NC, USA, 1989–2019) to investigate the relationships among street design, street network configuration, land-use and walking behaviour.

## 3. Analysis 

Two types of analyses were conducted to investigate the relative association of streetscape design –local qualities of street environment–, street network configuration –spatial structure of the urban grid– and land-use patterns with the distribution of flows. First, descriptive statistics were estimated for each area, summarising the averages of population/pedestrian densities, street network configuration, street-level attributes and land-uses. This allowed for illustrating the similarities and differences between the study areas as well as hinting to any existing general trend between the pedestrian densities and other attributes (e.g., land-use compositions). Second, multivariate regression analyses were conducted to examine the associations between street network configuration, streetscape design and street-level land-use characteristics in explaining the distribution of pedestrian densities. Linear models were developed both for all areas as considered as a single set and separately for individual areas. Three sets of models were constructed in the linear models. The first set of models includes land-use variables (land-use variables were entered into the regression first to allow for the evaluation of these variables in context relative to other factors affecting pedestrian behaviour). In the second and third sets of models, street network configuration and street-level design measures were entered respectively to understand the comparative effect and significance levels of each measure in explaining the distribution of pedestrian flows. Logarithmic transformation was applied to transform the distribution of flows into a normal distribution. Models were checked for multivariate regression assumptions (normality, constant variance and multi-collinearity) in JMP statistical software (SAS Institute, Cary, NC, USA). All results indicate that the models do not violate multivariate regression assumptions.

## 4. Findings

### 4.1. Gross Differences Between the Four Areas

Table 1 presents a quantitative profile of the selected areas in terms of population and pedestrian densities, configuration of street layouts, street-level pedestrian environments and land-uses at the ground-floor level. This preliminary benchmarking demonstrates notable differences between areas. The population densities of the areas, calculated on the basis of the census blocks associated with the street segments for which pedestrian counts were taken, range from 15 to 204 per hectare with Avcılar and Beylikdüzü having similar densities. The average number of moving pedestrians per 100 m is 52.7, 44.5, 54.1 and 128.4 for Beylikdüzü, Küçükçekmece, Büyükçekmece and Avcılar respectively. The four areas also differ significantly in their street configuration. Average 2-directional reach is highest for Beylikdüzü and lowest for Küçükçekmece. While Küçükçekmece and Avcılar have the highest average Integration, Beylikdüzü and Büyükçekmece have similar lower averages. In terms of street-level pedestrian attributes, all areas have approximately similar average sidewalk width, with Büyükçekmece and Küçükçekmece having the highest and lowest averages respectively. While average number of streets with trees and crosswalks are consistently low for Küçükçekmece, this area has the highest average presence of traffic lights. Küçükçekmece has the highest average number of residential uses per 100m. Average number of recreational uses per 100m is similar for all areas, except for Beylikdüzü, which has the highest average. Similarly, the average number of retail uses is comparable across Beylikdüzü, Büyükçekmece and Avcılar, while Küçükçekmece has the highest retail ground-floor activities. 

Overall, the initial tabulation suggests a correspondence between the average volume of pedestrian movement, street design and land development. However, since a sample of only four areas does not allow further statistical inference, linear models are developed in the next section to examine the associations further. 

### 4.2. Analysis of the Four Areas as a Single Set

Table 2 summarizes the results of regression models for three sets of models estimating the natural logarithm of pedestrians relativized by 100 m for all areas considered as a single set. Ground-floor land-use is found to explain more than ⅓ of the variation in the distribution of flows. The inclusion of structural measures and streetscape attributes results in similar levels of increase in the predictive power of the model (adj R^2^ change = 3–4%; *p* < 0.001) and the final urban form model can explain around 50% of the variation in pedestrian densities. By looking at the standardised Beta (std *β*) values, it can be argued that the number of recreational land-uses per 100 m is the most significant predictor of movement densities. In fact, the impact level and significance of this variable is quite consistent across the three sets of models. The effect levels of the number of retail activities per 100 m and 2-directional reach are similar (std *β* = 0.25–26, 99% CI), both being positively and significantly associated with the variation in pedestrian flows. In other words, increased retail uses opening onto a street, which has relatively higher directional accessibility, draws pedestrians within the surrounding network. Number of residential uses per 100 m is negatively associated (95% CI) with the pedestrian densities, which indicates that decreasing residential frontages and in turn increasing retail activities on the ground-floor, would significantly increase pedestrian movement densities. This finding is in conformity with recent research suggesting that pedestrian movement levels decrease with increased residential density [68,118,119]. For street design measures, the only significant correlate of movement density is the average sidewalk width. Surprisingly, no significant associations were found between the presence of crosswalks, traffic lights and trees along the segments and distribution of flows. This may be due to the fact that there is not enough variability among the selected areas in terms of this safety and aesthetics attributes. 

### 4.3. Analysis of Individual Areas

In order to better understand the distribution of pedestrians in each neighbourhood, multivariate regression models were estimated by considering individual areas separately. Table 3, Table 4, Table 5 and Table 6 demonstrate the results of regression models estimating the distribution of pedestrian densities for individual areas. 

In line with the previous overall model, the results suggest that the primary factors in explaining the distribution of pedestrian movement are the number of recreational land-uses at the street segment scale along with the configurational measure 2-directional reach. Even though Integration (r:n) does not appear as a significant variable, the inclusion of configurational measures, Integration and 2-directional reach, adds a considerable increase (adj R^2^ change = 12–20%, *p* < 0.001) to the explanatory powers of the models for each individual area, except for Küçükçekmece. 

Table 3 presents the individual impacts of land-use, street configuration and streetscape attributes on the distribution of movement in Avcılar. While street-level land-use explains 53% (99% CI) of the variation in pedestrian movement, the inclusion of spatial structural measures adds a 12% increase (*p* < 0.001) in the explanatory power of the model. Land-use and spatial structure variables together explain 65% (99% CI) of the variation in pedestrian movement. However, the inclusion of streetscape design measures does not add any significant improvement in the explanatory power of the overall model. In fact, none of the streetscape design attributes entered as significant measures.

Similarly, in Beylikdüzü (Table 4), the inclusion of street network measures adds a significant increase of 20% (*p* < 0.001) to the predictive power of the model, whereas, no significant increase in the explanatory power of the model is observed when streetscape design variables are added to the model. On the contrary, there was an inconsequential drop (2%, *p* < 0.01). Again, none of the street-level design attributes were found to be significantly correlated with pedestrian movement. 

More interestingly, similar to the findings reported in section ‘Analysis of the four areas as a single set’, the coefficient of 2-directional reach is statistically significant in all models, except for Küçükçekmece. Put simply, street segments that give more direct access to more surrounding streets draw greater volumes of pedestrians. In other words, pedestrians choose to walk on streets with increased directional accessibility provided by the straightness of street alignment. 

In Küçükçekmece, however, spatial variable 2-directional reach fails to correlate with pedestrian movement, as shown in Table 5. Land-use variables, on the other hand, are the strongest correlates of movement. Both the number of retail and recreational activities related with the street segment within this area is positively and significantly associated with the distribution of flows. This finding shows that the pedestrians in Küçükçekmece do not orient themselves according to the spatial structure of the network but are directed towards local attractors, such as various restaurants and shops. Hence, it appears that in Küçükçekmece land-use evolved in a manner that did not minimize travel distances from surrounding areas, and, as a result, street network configuration appears not to be a significant correlate of walking behaviour. 

In Büyükçekmece (Table 6), which has an even pattern of land-use dominated by residences, there is a moderate relationship between land-use and pedestrian movement (44%, *p* < 0.01). By including spatial structural measures in the model, the explanatory role of land-use in predicting movement density is significantly attenuated (adj R^2^ change = 17%; *p* < 0.001). Surprisingly, 2-directional reach is negatively correlated with movement. This contradictory result could be due to the fact that there is limited variability in the spectrum of this measure within the study area and that streets with increased directional accessibility are located in the outskirts of the neighbourhood, where observation points are few. Interestingly, when streetscape design variables are added, a modest increase of 9% (*p* < 0.001) is observed in the predictive power of the model. Average sidewalk width is the only variable that is significantly associated with the distribution of flows. In fact, in Büyükçekmece average sidewalk width has the highest effect level among all the variables. The weak correlation of this variable with movement in other areas might be due the fact that these neighbourhoods have more or less a uniform standard of sidewalk width. 

In conclusion, results reporting analyses for all areas considered as a single set are quite consistent with those obtained from the analyses of areas separately. Two macro-scale environmental variables –number of recreational uses at the street segment-level and 2-directional reach– are the most significant correlates of movement. Consistent with theory, movement densities are strongly associated with the number of recreational (i.e., parks) uses related to the individual segment. The above-results suggest that the impact of recreational land-use on the distribution of flows is quite consistent across models. The model developed considering all areas as a single set demonstrates the number of residential and retail frontages as significant correlates of walking. However, in the analysis of individual areas, these land-use measures are less consistently correlated with the distribution of flows. The consistent relation of 2-directional reach with pedestrian movement across models indicates that the manner in which streets are aligned and the direction changes needed to navigate the network also affect movement. This is consistent with findings reported in recent studies [77]. In Küçükçekmece, the network plays a secondary role in explaining the distribution of pedestrians. 

The evidence relating micro-scale environmental variables to walking is rather limited. While average sidewalk width is positively and significantly associated with pedestrian flows in the overall model (when all areas are analysed as a single set), a different picture emerges when areas are analysed separately. The significance of this variable is prevalent only in Büyükçekmece, which has a wider range of average sidewalk widths. On the other hand, no other consistent associations are found for the rest of the street design variables.

## 5. Discussion

In this research, linear models were developed to determine the extent to which streetscape design –local qualities of street environment–, street network configuration –spatial structure of the urban grid– and land-use patterns are associated with the distribution of walking within four peripheral neighbourhoods in İstanbul. Overall, the analyses presented suggest that local conditions within peripheral neighbourhoods are significantly related to walking behaviour. 

The results of this study emphasize the importance of including measures of street connectivity in physical activity and walkability studies. The results indicate that street connectivity is significantly associated with the distribution of pedestrian density over an area. This suggests that even after controlling for land-use and street-level design attributes, street layout plays a significant role in the way movement densities of pedestrians are distributed in the city. Street connectivity is measured through two syntactic measures, Integration and directional reach, which can capture the structural qualities of the street network. In all models, the standardized coefficient (stdβ) for directional reach, which measures the extent of streets captured within fewer direction changes, is positive and statistically significant (at a 95–99% level of confidence). This finding underscores the significance of the spatial structure of street networks, specifically the alignment of streets and the directional distance hierarchy engendered by the street network. This supports earlier findings arguing that street segments that give more direct access to more surrounding streets (accessible with fewer direction changes from the surroundings) tend to attract higher pedestrian flows [70,120].

In terms of local qualities of street environment, findings of this study are consistent with earlier findings arguing that sidewalk design provisions seem to be strong predictors of walking behaviour [121]. The models developed indicate that average sidewalk width is significantly and positively associated with movement densities over and above other urban design features. While street-level pedestrian indicators, such as the presence of crosswalks/traffic lights and tree-lined streets, are found to be significantly associated with walking [122], our data showed no significant relationships between these factors and pedestrian movement. 

The present study confirms past research findings that land-use is often associated with pedestrian movement. The regression models developed suggest that increased number of retail frontages is significantly associated with increased pedestrian volumes. This finding extends those of previous studies reporting that ease of pedestrian access to relatively higher nearby destinations is related particularly to walking [24]. Hence, it can be concluded that streets with increased retail fronts, such as cafes, banks, shops and other services, are usually more stimulating to the passer-by, attracting pedestrians from the immediate surroundings as well as further away areas. Particularly in peripheral areas characterised by pedestrian-oriented retail development on ground-floors (such as Küçükçekmece), retail use out-performs the effects of street network configuration. It is important to note that Küçükçekmece neighbourhood, which dates back to the end of the 19th century, evolved over time (as opposed to the other study sites, which were fully or partially planned). This is partly apparent in its street network layout, which is dominated by a curvilinear street network pattern that partially turns into cul-de-sacs. Therefore, it appears that in Küçükçekmece land-use evolved along the streets with relatively lower connectivity levels (in a manner that did not minimize travel distances from surrounding areas), and, as a result, street network configuration is no longer a significant correlate of walking behaviour in this area (i.e. notwithstanding the adverse location of shops, people still have to access these). Although this finding is opposed to the arguments of Space Syntax theory, it is indicative of the fact that history does not always follow regular rules.

The negative coefficients of the number of residences on the street segment indicate that movement levels decrease with higher residential frontages. The findings are somewhat in contrast to those indicating positive significant correlations between residential land-uses and walking behaviour [123,124]. This contrast among studies might be due in part to scale of measurement (e.g., most studies investigating the physical activity correlates of built environment measure residential activities in terms of densities or as a component of mixed land-use entropy index) or to the specific nature of İstanbul (e.g., in contrast to studies reporting on the US, where single zoning is dominant, in İstanbul the prevailing pattern consists of non-residential land-uses such as shops, cafes and banks located on the ground-floors of residences). In fact, recent studies conducted in different parts of İstanbul have reported similar results [113]. Thus, it can be suggested that in peripheral neighbourhoods walking behaviour can be enhanced by increasing retail activities and reducing residential uses on the ground-floors, thus increasing local attractors within the area.

In addition, stronger relationships are observed between the number of recreational land-uses and movement, suggesting public open space (e.g., playground, public parks) as a significant correlate of walking. In fact, the consistent positive effects of this indicator across the models (both all areas considered as a single set and four areas considered individually) contributes to past research, which presented ambiguous findings with regard to the associations of recreational land-uses and walking behaviour. Having greater number of recreational activities within peripheral districts might lead to increased densities of pedestrian movement, leading to greater levels of physical activity. 

### 5.1. Study Limitations

Our conclusions are tempered by several study limitations. First, the explanatory power of the linear model considering all areas as a single set (Table 2) displays a moderate degree of correlation, which indicates that there might be other factors (e.g., pedestrian perceptions regarding safety) influencing the distribution of pedestrian densities within the peripheral districts. However, it should be noted that the coefficients of correlations for all models considering individual areas separately (Table 3, Table 4, Table 5 and Table 6) show high correlations, ranging from 53% to 71%. Second, land-use is measured based on the number of frontages at the segment-level. Other studies have shown land-use density at the segment scale to be a significant correlate of movement as well [77]. Thus, future research needs to consider land-use at different measurement scales. However, recent research has also shown number of frontages on ground-floors to be more significantly correlated with pedestrian movement than land-use densities at the segment scale [113]. Third, the sample is based on four areas with similar peripheral characteristics in a single city. Hence, these results need to be tested in other peripheral areas of various metropolitan areas to see whether these results are generalizable to other peripheral urban conditions. Fourth, the study areas lacked heterogeneity across some of the measures used, such as the sidewalk widths. This might be one of the reasons why no significant relationships are identified between street-scale attributes and walking. More research conducted within more heterogeneous urban environments is needed to shed more light into this association. Furthermore, pedestrian observations were conducted for a limited duration (10-min intervals on two different weekdays on mornings and afternoons) on a limited number of segments (~30 per area), which might have tempered the results. Since the use of advanced data collection technologies, such as video cameras and position sensors, is not yet prevalent in Turkey, a limited amount of data could be captured manually. Further research that includes pedestrian counts taken at varying times, various durations and along an increased number of segments through automated systems might more clearly detect relationships with walking. In addition, because the analysis is cross-sectional, the relationships observed do not necessarily indicate causality. Future work is needed to develop an understanding of how micro- and macro-scale street-level attributes are interrelated and how they are associated with walking relative to other environmental (e.g., aesthetics and comfort such as tree-aligned streets) and social features (e.g., future research should include both perceived and objective measures, particularly for characteristics such as safety [23,125,126,127]. Finally, there is an inherent limitation to this research and similar studies: they do not consider whether people enjoy walking or not. For example, through observation and surveys Mehta [128] found that some attributes of the street environment, such as the presence of other people, multiple activities and street width, added to the sensory pleasure of the street. The activity of walking is definitely important for public health professionals, yet the pleasure of walking is also important for city planners and urban designers. Thus, future research, such as Mehta’s [129] and Darker et al.’s [130], should also take into account the perceptions of pedestrians in different neighbourhoods.

Research in these areas is fairly limited; hence, this study contributes to the literature by investigating the comparative roles of macro-scale (land-use and street design) and micro-scale (pedestrian-friendly street design) environmental correlates of walking. The results of the present study also demonstrate the significance of a more recent walkability indicator, directional reach, which can provide alternative means to quantify street design and capture a subtler relationship between movement patterns and urban systems. 

### 5.2. Practical Implications

Important implications arise from the findings on the significance of street network design, average sidewalk width and ground-floor frontages in explaining the distribution of walking within peripheral areas. These findings also add support to calls for policy initiatives to create more-walkable neighbourhoods at the periphery [23,131]. One practical implication of these findings would be the provision of more generous sidewalks on spatially more prominent streets (i.e. streets with higher directional accessibility provided by the straightness of street alignments) in the light of the association between measures of the built environment and movement densities. The consistent association of land-use patterns with the distribution of movement shown in this study is critical for urban designers since it points to the fact that the strategic design of the ground-floor at the road segment scale is essential in designing for urban vitality and enhanced physical activity. Findings from this study also suggest that public health, urban design and planning strategies and intervention programmes to promote walking need to consider the contribution of street-level (both micro- and macro-scale) built environment factors to facilitate physical activity among communities.

## 6. Conclusions

City planning and healthy policy agendas to promote physical activity or walking need to emphasize the crucial role of micro- and macro-level environmental attributes that facilitate opportunities for people to be more active. In this respect, findings from this study suggest that peripheral neighbourhoods containing more direct and linear streets with extensive sidewalks, greater amounts of open green spaces for recreation and increased numbers of retail frontages, are likely to be supportive of walkable neighbourhoods. Since the results show the relative contribution of each of these environmental characteristics to walking, the results can lead to evidence-based policies and programs aimed at increasing walking and hence physical activity. For example, urban designers presume that pedestrian-oriented street-level features are important for active street life but they have little empirical evidence to back their claim. Hence, the results of this study underlie the argument that the layout of suburbs and traditional urban neighbourhoods is insufficient to encourage walking over automobile use and that a number of factors, including the location of retail stores and pedestrian-oriented commercial buildings, are required [132,133].

## Figures and Tables

**Table 1:** Characteristics of selected areas summarised in terms of population and pedestrian densities, street network configuration, street design and land-use.

Variable	Beylizdüzü	Küçükcekmece	Büyükçekmece	Avcılar	All
Densities of residential Population and pedestrians					
population density per hectare	84.57	204.19	15.45	104.11	102.08
average number of pedestrians per 100 m	52.72	44.51	54.14	128.41	69.94
Segment-level street configuration					
avg 2-Directional Reach (20^o^)	480.92	192.77	241.85	317.16	307.25
avg Integration (*n*)	0.79	0.95	0.71	0.96	0.85
Street-level pedestrian environment					
avg.sidewalk width	165.12	109.62	196.3	167.06	160.2
tree presence [yes]	43%	7%	53%	59%	49%
crosswalk presence [yes]	20%	7%	13%	2%	12%
traffic light presence [yes]	7%	10%	4%	3%	6%
Street-level land-use					
avg # residential use per 100 m	1.46	7.63	3.49	5.40	4.37
avg # recreational use per 100 m	0.62	0.36	0.37	0.36	0.44
avg # retail use per 100 m	0.90	2.91	1.16	1.20	1.36

**Table 2:** Multivariate regression for three sets of models estimating the distribution of pedestrian flows for all areas considered as a single set. “Land-use” shows the effects of solely land-use variables on the distribution of flows. “Land-use+spatial structure” and “Urban Form” show the effects of adding street network configuration and street-level design measures into the model respectively.

Variable	Land-Use			Land-Use+Spatial Structure			Urban Form		
	*β*	*t*	*std β*	*β*	*t*	*std β*	*β*	*t*	*std β*
Street-level Land use (ground-floor)									
# residential-use per 100m	−0.07 ***	−3.53	−0.28	−0.06 **	−3.16	−0.24	−0.05 *	−2.47	−0.20
# retail-use per 100m	0.12 ***	4.12	0.31	0.12 ***	4.13	0.31	0.10 **	3.33	0.26
# recreational-use per 100m	0.57 ***	6.14	0.46	0.62 ***	6.82	0.50	0.67 ***	7.32	0.54
Street network configuration									
Integration (n)				−0.00 *	−2.06	−0.15	−0.00	−1.05	−0.08
2-Directional Reach (20°)				0.00 **	3.15	0.23	0.00 ***	3.40	0.25
Street-level design									
crosswalk presence [yes]							-0.11	0.80	−0.06
traffic light presence [yes]							-0.24	1.27	0.10
average sidewalk width							0.00 **	2.96	0.23
presence of trees [yes]							0.07	0.86	0.06
Number of observations: 120									
Adj R-squared	0.38 ***			0.42 ***			0.45 ***		

*** *p* < 0.001; ** *p* < 0.01; * *p* < 0.05 (two-tailed tests).

**Table 3:** Multivariate regression for three sets of models estimating the distribution of pedestrian flows in Avcılar. “Land-use” shows the effects of solely land-use variables on the distribution of flows. “Land-use+spatial structure” and “Urban Form” show the effects of adding street network configuration and street-level design measures into the model respectively.

Variable	Land-Use			Land-Use+Spatial Structure			Urban Form		
	*β*	*t*	*std β*	*β*	*t*	*std β*	*β*	*t*	*std β*
Street-level Land use (ground-floor)									
# residential-use per 100m	−0.23 ***	−3.85	−0.54	−0.17 **	−2.79	−0.40	−0.15 *	−2.16	−0.35
# retail-use per 100m	0.24 *	2.39	0.33	−0.14	−0.94	−0.19	−0.15	−0.94	−0.20
# recreational-use per 100m	0.30	1.29	0.19	0.46 *	2.04	0.29	0.58 *	2.16	0.37
Street network configuration									
Integration (n)				0.00	0.39	0.06	0.00	0.14	0.02
2-directional reach (20^o^)				0.00 **	2.93	0.58	0.00 **	3.04	0.63
Street-level design									
traffic light presence [yes]							−0.23	0.43	0.06
average sidewalk width							0.00	1.22	0.17
presence of trees [yes]							0.11	0.52	0.08
Number of observations: 30									
Adj R-squared	0.53 ***			0.65 ***			0.63 ***		

*** *p* < 0.001; ** *p* < 0.01; * *p* < 0.05 (two-tailed tests).

**Table 4:** Multivariate regression for three sets of models estimating the distribution of pedestrian flows in Beylizdüzü. “Land-use” shows the effects of solely land-use variables on the distribution of flows. “Land-use+spatial structure” and “Urban Form” show the effects of adding street network configuration and street-level design measures into the model respectively.

Variable	Land-Use			Land-Use+Spatial Structure			Urban Form		
	*β*	*t*	*std β*	*β*	*t*	*std β*	*β*	*t*	*std β*
Street-level Land use (ground-floor)									
# residential-use per 100m	−0.14	−1.27	−0.19	−0.05	−0.50	−0.07	0.08	0.52	0.10
# retail-use per 100m	0.08	1.18	0.18	0.09	1.43	0.19	0.07	1.07	0.16
# recreational-use per 100m	0.54 ***	3.75	0.58	0.57 ***	4.14	0.61	0.51 **	3.21	0.55
Street network configuration									
Integration (n)				0.00	0.68	0.12	0.00	1.02	0.24
2-directional reach (20^o^)				0.00 **	2.68	0.41	0.00 *	2.21	0.38
Street-level design									
crosswalk presence [yes]							0.17	0.82	0.15
traffic light presence [yes]							−0.34	−1.07	−0.17
average sidewalk width							−0.00	−0.60	−0.12
presence of trees [yes]							0.05	0.31	0.05
Number of observations: 31									
Adj R-squared	0.35 **			0.55 ***			0.53 **		

*** *p* < 0.001; ** *p* < 0.01 (two-tailed tests).

**Table 5:** Multivariate regression for three sets of models estimating the distribution of pedestrian flows in Küçükçekmece. “Land-use” shows the effects of solely land-use variables on the distribution of flows. “Land-use+spatial structure” and “Urban Form” show the effects of adding street network configuration and street-level design measures into the model respectively.

Variable	Land-Use			Land-Use+Spatial Structure			Urban Form		
	*β*	*t*	*std β*	*β*	*t*	*std β*	*β*	*t*	*std β*
Street-level Land use (ground-floor)									
# residential-use per 100m	−0.01	−0.66	−0.08	−0.03	−1.38	−0.21	−0.01	−0.49	−0.08
# retail-use per 100m	0.09 ***	3.91	0.43	0.12 ***	3.79	0.57	0.13 **	3.61	0.64
# recreational-use per 100m	0.91 ***	7.24	0.78	0.86 ***	6.36	0.74	0.92 ***	6.28	0.78
Street network configuration									
Integration (n)				0.00	−1.11	−0.17	−0.00	−1.09	−0.17
2-directional reach (20^o^)				-0.00	−0.40	−0.05	−0.00	−0.17	−0.03
Street-level design									
crosswalk presence [yes]							−0.37	−1.31	−0.18
traffic light presence [yes]							0.36	1.76	0.24
average sidewalk width							−0.00	−0.46	−0.07
presence of trees [yes]							−0.10	−0.76	−0.09
Number of observations: 29									
Adj R-squared	0.71 ***			0.71 ***			0.71 ***		

*** *p* < 0.001; ** *p* < 0.01 (two-tailed tests).

**Table 6:** Multivariate regression for three sets of models estimating the distribution of pedestrian flows in Büyükçekmece. “Land-use” shows the effects of solely land-use variables on the distribution of flows. “Land-use+spatial structure” and “Urban Form” show the effects of adding street network configuration and street-level design measures into the model respectively.

Variable	Land-Use			Land-Use+Spatial Structure			Urban Form		
	*β*	*t*	*std β*	*β*	*β*	*t*	*std β*	*t*	*β*
Street-level Land use (ground-floor)									
# residential-use per 100m	−0.14 **	−2.81	−0.40	−0.13 **	−3.02	−0.37	−0.14 **	−3.58	−0.39
# retail-use per 100m	0.20 **	2.88	0.40	0.23 **	3.48	0.45	0.15	1.97	0.29
# recreational-use per 100m	0.35 *	2.15	0.31	0.23	1.66	0.21	0.26 *	2.05	0.23
Street network configuration									
Integration (n)				0.01 **	2.76	0.36	0.01	1.56	0.20
2-directional reach (20^o^)				−0.00 **	−2.93	−0.38	−0.00B *	−2.10	−0.26
Street-level design									
crosswalk presence [yes]							0.21	1.07	0.14
traffic light presence [yes]							–	–	–
average sidewalk width							0.00 *	2.38	0.33
presence of trees [yes]							−0.05	−0.42	0.04
Number of observations: 31									
Adj R-squared	0.44 **			0.61 ***			0.70 ***		

*** *p* < 0.001; ** *p* < 0.01; * *p* < 0.05 (two-tailed tests).
